# The role of CT myelography in sparing the spinal cord during definitive radiotherapy in vertebral hemangioma

**DOI:** 10.1002/acm2.12144

**Published:** 2017-08-18

**Authors:** SP Sudha, MS Gopalakrishnan, K Saravanan

**Affiliations:** ^1^ Department of Radiotherapy Jawaharlal Institute of Post‐Graduate Medical Education and Research (JIPMER) Pondicherry India; ^2^ Department of Neurosurgery Jawaharlal Institute of Post‐Graduate Medical Education and Research (JIPMER) Pondicherry India; ^3^ Department of Medical Physics Jawaharlal Institute of Post‐Graduate Medical Education and Research (JIPMER) Pondicherry India

**Keywords:** CT myelography, susceptibility artifacts

## Abstract

**Introduction:**

The spinal cord is poorly visualized on CT images but is well visualized in MRI images. However, implants used for spinal stabilization can produce artifacts on the MRI images which can interfere with identification of the cord. CT myelography in conjunction with CT simulation helps to clearly delineate the cord.

**Materials and methods:**

CT simulation was done in a patient with vertebral hemangioma. Pre‐ and post myelography images were obtained. Two plans were generated on pre and post myelography CT images using Eclipse™ treatment planning system (TPS) version 10.0, Varian Medical Systems, USA. The prescribed total dose to PTV was 40 Gy in 20 fractions.

**Results:**

The cord was poorly visualized in the premyelogram CT images. The titanium implants used for spinal cord stabilization produced artifacts in the MRI images. Figure 1 Post myelogram, the contrast lit up the spinal cord.

There was an overlap of 0.75 cc volume of the spinal cord with the PTV in the premyelogram images. This volume was reduced to 0 cc in the post myelogram images. There was an overlap of 5.4 cc volume of the PRV of spinal cord with the PTV in the premyelogram images which was reduced to 1 cc in the post myelogram images. The overlap region between the PTV and spinal cord received around 71% of the prescribed dose in premyelogram CT could be reduced to 0% in the post myelogram CT. The mean dose received by the overlap PRV spinal cord and PTV could be increased from 70% in the premyelogram to 92% in the post myelogram plans.

**Conclusion:**

CT myelogram in conjunction with CT simulation is particularly useful in cases where the tumor margin is very close to the cord and spinal implants are causing distortion of magnetic resonance images.

## 1 INTRODUCTION

With the availability of high‐precision radiotherapy techniques it is possible to target tumor accurately and minimize the doses to surrounding critical structures. For tumors in and around the spinal cord it is imperative that we delineate the cord precisely.

The spinal cord is poorly visualized on CT images. Traditionally, it is the spinal canal which is often contoured as the spinal cord in CT scans. Spinal cord can be accurately contoured on the registered MR images. However, if implants are used for spinal stabilization it can produce artifacts on the MRI images which can interfere with identification of the cord accurately.^1^, ^2^ Sometimes the tumors involving the vertebral bodies per se also hinder identification of the cord.

It is of paramount importance to identify the cord accurately and limit the dose to the cord, since excessive dose to the spinal cord is associated with irreversible myelopathy.^3^ CT myelography is a technique which is used to identify abnormalities in the spinal cord in cases where MRI is contraindicated. We decided to do a CT myelogram in conjunction with CT simulation in a patient with vertebral hemangioma and we report the differences in the pre and post myelogram planning parameters to highlight the advantage of combining this technique.

## 2 MATERIALS AND METHODS

The patient was immobilized using a Vac‐Lok [manufacturer ‐Klarity medicals] in the supine position. CT simulation was taken using CT Somatom Spirit a dedicated CT machine for simulation. CT was acquired from T6 to L5. Our CT scanner does not have a metal artifact reduction algorithm.

Images acquired from CT had a slice thickness of 3 mm and the reconstructed image from the Eclipse registration software was also 3 mm. After premedication a lumbar puncture was done, dye was injected into the spinal canal and the patient was immobilized using the same Vac‐Lok. The neurosurgeon performed the lumbar puncture and injection. No additional staff was required for the simulation. The normal CT simulation consent form was modified to inform the patient about any potential risks of CT myelography.

Premedications for myelogram and post myelogram instructions were given. However, the procedure of CT simulation remained the same.

A second set of simulation images were taken using the same parameters. Two volumes were contoured – Gross tumor volume and Planning target volumes. Spinal cord, kidneys, lungs, stomach and intestines were the organs at risk. Contouring was done separately for each CT image. The tumor in both the instances was drawn based on the registered MRI images. However in Plain CT images, the area of the tumor drawn was an approximation since the cord was not seen due to the susceptibility artifacts in the fused MR images. However in the post myelogram CT images, the tumor edges could be drawn clearly where it was seen distinct from areas occupied by the contrast in the spinal canal and the cord. This led to a reduction in the PTV volume by almost 20 cc.

The spinal cord is suspended in the CSF and is tethered by roots and the dentate ligament. Hence it is unlikely to move significantly in the spinal canal on a day to day basis. However, small movements were accounted for by giving a small margin around the spinal cord of 3 mm. We gave a margin of 3 mm around the cord to create the PRV cord [planning organ at risk volume].

Plans were generated on pre‐ and post myelography CT images using Eclipse™ treatment planning system (TPS) version 10.0, Varian Medical Systems, USA. The TPS used analytical anisotropic algorithm (AAA) algorithm for calculation of monitor units and progressive resolution optimizer (PRO) version 10 for VMAT plans. Since the contouring was done separately for each CT image and there was a difference in the PTV volume between the two images, separate plans had to be created for the pre and post myelogram images.

During planning, the contrast around the cord had a Hounsfield unit (HU) of 200–350 HU. We have assigned HU manually for the contrast. Since the spinal canal contains CSF and during the actual treatment it is the CSF which fills the spinal canal, we assigned tissue equivalent value for the contrast which is zero HU.

The prescribed total dose to PTV was 40 Gy in 20 fractions and the optimization constraint was that 95% isodose line encompasses 95% of PTV. The optimization constraint given for the spinal cord was that the D‐Max dose to any part of the spinal cord was kept below 40 Gy and both the plans met the specified objectives.

The Varian Clinac iX with 6 MV photon energy was used for delivering the treatment.

## 3 RESULTS

**Figure 1:**
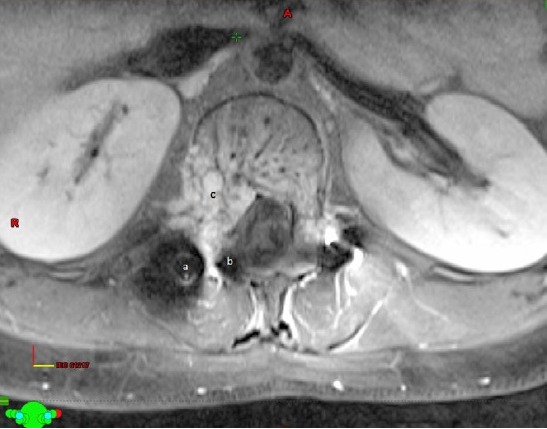
(a) Titanium implant, (b) Susceptibilty artifact encroaching into the spinal canal, (c) Contrast enhancing tumor.

**Figure 2:**
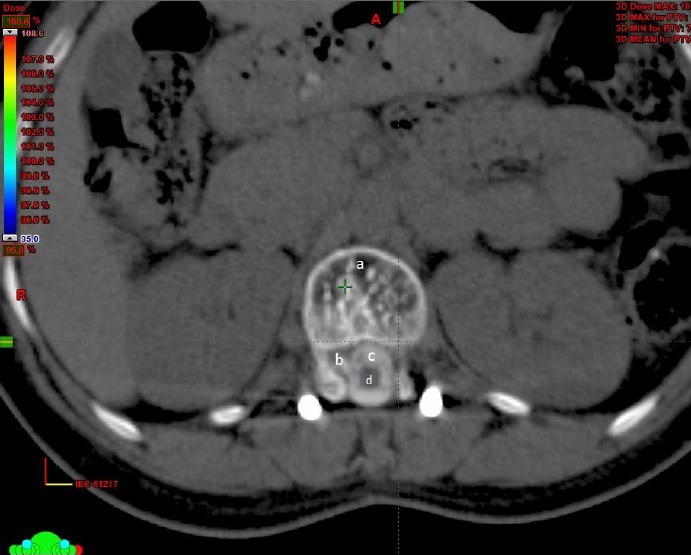
(a) Typical polka dot appearance of a hemangioma (b) Extension of the hemangioma close to the spinal canal (c) Myelogram 2d‐Spinal cord.

**Figure 3:**
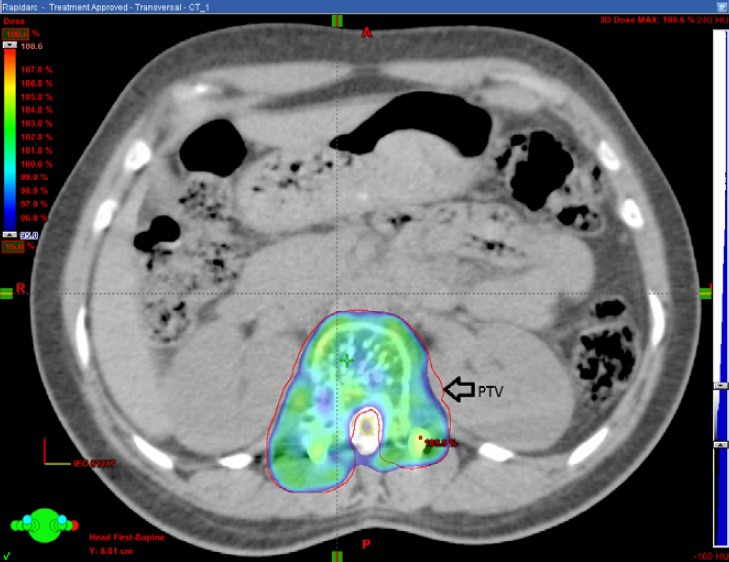
Outline of the PTV on CT myelogram.

The cord was poorly visualized in the premyelogram CT images. The titanium implants used for spinal cord stabilization produced artifacts in the MRI images. Figure 1 This made it difficult to clearly visualize the cord on the registered MRI images. Post myelogram, the contrast lit up the spinal cord which then could be clearly delineated Figs. 2 and 3.

Contouring was done separately for each CT image. There was a difference in the PTV volume between the two images. The volume of PTV CT myelogram was 128.38 cc and PTV CT plain was 140.42 cc.

There was an overlap of 0.75 cc volume of the spinal cord with the PTV in the premyelogram images, which was reduced to 0 cc in the post myelogram images. There was an overlap of 5.4 cc volume of the PRV of spinal cord with the PTV in the premyelogram images which was reduced to 1 cc in the post myelogram images.

The dosimetric parameters in percentage and absolute value in Gy are outlined in Table 1. The maximum dose received by the PTV was 108% in both the plans The D‐mean in both the plans were also 100%. This ensured comparability of the data collected between the two plans.

**Table 1:** Dosimetric parameters

Parameters	Premyelogram	Post myelogram
PTV volume	140.42 cc	128.38 cc
Overlap cord‐PTV	0.75 cc	0
Overlap PRV cord‐PTV	5.4 cc	1 cc
PTV		
D‐max	108%	108%
D‐mean	100%	100%
Spinal cord		
D‐max	88.4% [35.36 Gy]	84% [33.6 Gy]
D‐mean	52%[20.8 Gy]	27%[10.8 Gy]
PRV‐spinal cord		
D‐max	108%[43.2 Gy]	101.8%[40.7 Gy]
D‐mean	69%[35.36 Gy]	33.7%[35.36 Gy]
Overlap spinal cord and PTV		
D‐mean	71%[28.4 Gy]	0%
		No overlap between spinal cord and PTV in post myelogram CT
Overlap PRV‐Spinal cord and PTV		
D‐mean	70%[28 Gy]	92%[36.8 Gy]
		Dose could be increased to the overlap region between PRV‐spinal cord and PTV

The D‐max received by the cord in the pre‐ and post myelogram images are 88.4% and 84%, respectively. The D‐mean received by the cord reduced by half from 52% in the premyelogram image to 27% in the post myelogram image.

The D‐max received by the PRV cord in the pre‐ and post myelogram images are 108% and 101.8%, respectively. The D‐mean received by the PRV cord reduced by half from 69% to 33.7%.

The overlap region between the PTV and spinal cord received around 71% of the prescribed dose. This was reduced to 0% in the post myelogram CT. However, the mean dose received by the overlap PRV‐spinal cord and PTV could be increased from 70% the premyelogram CT to 92% in the post myelogram plans.

We have also applied the same plan done on the premyelogram CT to the post myelogram CT.

The only complication which was reported was headache which developed 24 hr after the procedure. Headache lasted for 1 day and subsided with adequate hydration, paracetamol and foot end elevation.

## 4 DISCUSSION

Radiation damage to the spinal cord is one of the most dreaded complications in the treatment of cancer.^4^ Very often, dose to the tumor is compromised to avoid overdosing the cord.^5^ Contouring the cord in CT involves drawing the entire spinal canal or a circular hypodense area within the spinal canal. The PRV of 3 mm is given to this volume and as per most guidelines the dose to this area is kept below 50 Gy and dose to the spinal cord is kept below 45 Gy in conventional fractionation.

When the tumor is very close to the cord often the dose to the tumor is compromised to spare the cord. This will inevitably result in recurrence of the tumor. Hence, it is imperative that the cord is identified as accurately as possible in such cases and not use the spinal canal volume as a surrogate for the cord. Registration with MRI and contouring the cord on the registered MRI is often the solution. However, this has its own errors especially due to the artifacts caused by the implants used for spinal stabilization. Titanium implants can cause distortion of the image or even obscure surrounding anatomy around them due to susceptibility artifacts. Figure 1 In such cases, we suggest that identification of the cord is done by CT myelogram.

In our patient, the cord was poorly visualized in the plain CT images. We could not reliably contour the cord using the registered MR images because of the severe distortion caused by the implants. Figure 1 Hence, we did a CT myelogram. The contrast formed a halo around the spinal cord which enabled us to visualize the cord clearly Figs. 2 and 3.

We wanted to see if there was an impact on the planning and dosimetric parameters. Identifying the cord clearly helped to decrease the overlap between the PTV and the cord and PRV cord. In the premyelogram images this overlap between PTV and cord was 0.75 cc and could be reduced to zero in the post myelogram CT images.

Similarly, the PRV cord volume also decreased from 5.4 to 1 cc. The mean dose received by the PTV and PRV cord overlap increased from 70.8% to 92%.

It is clear from our data that the dose to the overlap region was more in the post myelogram CT which indicates that this area which can potentially harbor tumor tissue could receive a higher dose. Thus, CT myelogram helped us to increase the dose to the PTV and decrease the dose to the spinal cord.

Thariat et al. studied the value of CT myelography in the delineation of the spinal cord and found that CT myelography had superior resolution to MRI when Cyber knife stereotactic radiotherapy was used.^6^ We compared the premyelogram and post myelogram plans in our patient where susceptibility artifacts were prominent. An important finding in our study was that dose to the overlap region [PTV and PRV cord] was more in the post myelogram CT, which indicates that this area which can potentially harbor tumor tissue could receive a higher dose.

As far as we know this is the first description of the use of CT myelogram to achieve dose optimization in radiation therapy of vertebral hemangioma in cases where spinal implants have been used.

We wanted to describe a simple method which will identify the cord accurately which is applicable in cases such as this where titanium implants obscured the normal anatomy. Since we could identify the cord clearly we could reduce the radiation dose well below the recommended tolerance dose of 40 Gy while keeping an optimal PTV dose. Even the recommended tolerance dose of 40–44 Gy to spinal cord has a 5 yr probability of complications of 5%. By staying below this dose, we reduce even this small probability of complication and such a strategy may reduce the risks of myelopathy in case the patient requires re‐irradiation in the future. An important finding in our study was that dose to the overlap region was more in the post myelogram CT, which indicates that this area which can potentially harbor tumor tissue could receive a higher dose. Thus, CT myelogram not only helped us to decrease the dose to the spinal cord but also to increase the dose to the PTV.

However, myelogram is contraindicated if there is a spinal block or if the cord is low lying. One should also be absolutely certain that only non‐ionic contrast is used since inadvertent intrathecal injection of ionic contrast media is fatal.^7^

## 5 CONCLUSION

CT myelogram in conjunction with CT simulation helps clearly visualize the cord. The technique we have described is particularly useful in cases, where the tumor margin is very close to the cord and spinal implants are causing distortion or obscuration of magnetic resonance images. Precise identification of the cord enabled us to deliver more dose to the tumor.

## CONFLICT OF INTERESTS

The authors declare no conflict of interest.
